# Supplementary material for: Asynchronous magnetic resonance elastography: Shear wave speed reconstruction using noise correlation of incoherent waves
Source: Magn Reson Med. 2022 Oct 27;89(3):990–1001. doi: 10.1002/mrm.29502 (PMC9792433; doi:10.1002/mrm.29502)
Supplement: Supplementary file 1 — DATA S1 MATLAB code used to generate the numerical simulations of Figure 1 [file MRM-89-990-s002.zip › k-Wave/helpfiles/focus.html]

focus :: Functions (k-Wave)


# focus

Create input signal based on source mask and focus position.

## Syntax

```
input_signal_mat = focus(kgrid, input_signal, source_mask, focus_position, sound_speed)
```

## Description

`focus` takes a single input signal and a source mask and creates an input signal matrix (with one input signal for each source point). The appropriate time delays required to focus the signals at a given position in Cartesian space are automatically added based on the user inputs for `focus_position` and `sound_speed`.

## Inputs

|  |  |
| --- | --- |
| `kgrid` | k-Wave grid object returned by `kWaveGrid` |
| `input_signal` | single time series input |
| `source_mask` | matrix specifying the positions of the time varying source distribution (i.e., source.p\_mask or source.u\_mask) |
| `focus_position` | position of the focus in Cartesian coordinates |
| `sound_speed` | scalar sound speed |

## Outputs

|  |  |
| --- | --- |
| `input_signal_mat` | matrix of time series following the source points using MATLAB's column-wise linear index ordering |
